# Using Aggregate Vasoactive-Inotrope Scores to Predict Clinical Outcomes in Pediatric Sepsis

**DOI:** 10.3389/fped.2022.778378

**Published:** 2022-03-04

**Authors:** Palak Shah, Tara L. Petersen, Liyun Zhang, Ke Yan, Nathan E. Thompson

**Affiliations:** ^1^Section of Critical Care Medicine, Department of Pediatrics, Medical College of Wisconsin, Milwaukee, WI, United States; ^2^Section of Quantitative Health Sciences, Department of Pediatrics, Medical College of Wisconsin, Milwaukee, WI, United States

**Keywords:** pediatric sepsis, sepsis outcomes, sepsis prediction, inotropic support, vasoactive-inotropic score

## Abstract

**Objectives:**

The heterogeneity of sepsis makes it difficult to predict outcomes using existing severity of illness tools. The vasoactive-inotrope score (VIS) is a quantitative measure of the amount of vasoactive support required by patients. We sought to determine if a higher aggregate VIS over the first 96 h of vasoactive medication initiation is associated with increased resource utilization and worsened clinical outcomes in pediatric patients with severe sepsis.

**Design:**

Retrospective cohort study.

**Setting:**

Single-center at Children's Wisconsin in Milwaukee, WI.

**Patients:**

One hundred ninety-nine pediatric patients, age less than 18 years old, diagnosed with severe sepsis, receiving vasoactive medications between January 2017 and July 2019.

**Interventions:**

Retrospective data obtained from the electronic medical record, calculating VIS at 2 h intervals from 0–12 h and at 4 h intervals from 12–96 h from Time 0.

**Measurements:**

Aggregate VIS derived from the hourly VIS area under the curve (AUC) calculation based on the trapezoidal rule. Data were analyzed using Pearson's correlations, Mann-Whitney test, Wilcoxon signed rank test, and classification, and regression tree (CART) analyses.

**Main Results:**

Higher aggregate VIS is associated with longer hospital LOS (*p* < 0.0001), PICU LOS (*p* < 0.0001), MV days (*p* = 0.018), increased in-hospital mortality (*p* < 0.0001), in-hospital cardiac arrest (*p* = 0.006), need for ECMO (*p* < 0.0001), and need for CRRT (*p* < 0.0001). CART analyses found that aggregate VIS >20 is an independent predictor for in-hospital mortality (*p* < 0.0001) and aggregate VIS >16 for ECMO use (*p* < 0.0001).

**Conclusions:**

There is a statistically significant association between aggregate VIS and many clinical outcomes, allowing clinicians to utilize aggregate VIS as a physiologic indicator to more accurately predict disease severity/trajectory in pediatric sepsis.

## Introduction

Sepsis is a leading cause of pediatric morbidity and mortality, accounting for 80,000 pediatric hospitalizations yearly in the U.S. alone with ~5,000 deaths and 25–40% of patients suffering long term complications (1–4). The highest risk of in-hospital mortality secondary to refractory septic shock occurs within the first 48–72 h of sepsis recognition or ICU admission (5, 6). For pediatric septic shock patients who survive, a recent multi-center study found that 35% of survivors had significant health-related quality of life deterioration from baseline at 12 months following hospitalization (7).

A clinical predictive tool utilizing a physiologic biomarker to assess pediatric severe sepsis in real time may be beneficial in clinical decision making. Vasoactive medications are one of the primary treatments for sepsis-related cardiovascular dysfunction and fluid-refractory septic shock (1, 3, 8). The vasoactive-inotrope score (VIS), derived from the inotrope score (IS), is a quantitative measure of the amount of vasoactive support required by patients (Supplementary Figure 1) (9). The application of VIS in pediatric sepsis, however, is limited to date (10, 11). Appreciating that the highest risk of mortality is within the first 48–72 h of sepsis identification or ICU admission, it would be beneficial to study a cohort over a longer duration of time, such as 96 h (5, 6). It would also be beneficial to study the correlation of the total inotropic demand over this time period as there is currently no literature on a 96-h aggregate VIS and its relationship to clinical outcomes in pediatric patients diagnosed with critical sepsis, severe sepsis, or septic shock.

The purpose of this study was to determine if a higher aggregate VIS over the first 96 h of vasoactive medication initiation is associated with increased resource utilization and worsened clinical outcomes, including longer PICU and hospital lengths of stay (LOS), increased mechanical ventilation (MV) days, and increased risk of in-hospital complications, including mortality, cardiac arrest requiring compressions, need for extracorporeal membrane oxygenation (ECMO), and need for continuous renal replacement therapy (CRRT) in pediatric patients with severe sepsis. A secondary aim of the study was to compare the results of aggregate VIS at a 96 h assessment point to previously published results at a single point in time at 48 h in an effort to determine the optimal time point for using VIS as a severity of illness tool in sepsis (11).

## Materials and Methods

A single-center retrospective chart review at Children's Wisconsin (CW), a free-standing quaternary-care children's hospital, was performed for pediatric patients diagnosed with severe sepsis. Study approval was obtained from the Children's Wisconsin Institutional Review Board (IRB). Retrospective data were collected from January 1, 2017 to July 31, 2019. Severe sepsis was defined as sepsis with cardiovascular dysfunction, acute respiratory distress syndrome (ARDS), dysfunction in 1 or more organ systems, and/or persistent hemodynamic instability despite initial fluid resuscitation therapy (Supplementary Table 1) (2–4, 8). For the purpose of this study, severe sepsis also included critical sepsis and septic shock diagnoses. Patients at CW triggered an electronic clinical sepsis alert based on these definitions. Time 0 was then established as the time of initiation of vasoactive medication rather than the clinical sepsis alert to appropriately capture and standardize this patient cohort as some patients triggered the clinical sepsis alert several hour to days before requiring vasoactive medications.

Patient electronic medical records were initially identified by the CW clinical sepsis alert and filtered for those patients who received vasoactive medications for the treatment of severe sepsis during their hospitalization (12, 13). Charts were then manually reviewed to determine if patients met the appropriate inclusion criteria. Inclusion criteria included age 0–17 years at the time of diagnosis, pediatric ICU admission, Time 0 within the study period, initial encounter for severe sepsis during the hospitalization, and vasoactive medications for a severe sepsis diagnosis (Supplementary Table 2). Data collection included patient demographics, laboratory data, aggregate VIS calculated over 96 h, total ICU and hospital lengths of stay, total mechanical ventilation duration, in-hospital mortality, cardiac arrest defined as requiring chest compressions for any length of time, total ECMO duration, and total CRRT duration. The Pediatric Risk of Mortality III (PRISM-III) scores, a third-generation physiology-based predictive score for pediatric risk of mortality, was also collected (14). Laboratory data included highest lactate level, though the source of laboratory draws (arterial, venous, or capillary) was not collected or specified. VIS was calculated at 2-h intervals from hour 0–12 and at 4-h intervals from h 12–96 to better capture frequent vasoactive medication titrations in a patient's initial clinical course. Titration of vasoactive medications were performed by clinician discretion based on available physiologic data. The 96-h timeframe was chosen to extend a day beyond the time of peak mortality from sepsis (6). Chart data were then cross-referenced with the Virtual PICU Systems, LLC (VPS^LLC^) database, an international collaborative including over 200 ICUs focused on standardizing pediatric critical care data for quality improvement and research. Data obtained through VPS^LLC^ included ICU and hospital LOS, invasive mechanical ventilation duration, ECMO duration, CRRT duration, and PRISM-III scores and risk of mortality, which helped ensure accurate data collection. These data were then imported into a Research Electronic Data Capture (REDCap) database for ease of review and analysis.

Aggregate VIS was derived from the hourly VIS area under the curve (AUC) calculation based on the trapezoidal rule. Data were analyzed using Pearson's correlations, Mann-Whitney test, Wilcoxon signed rank test, and classification and regression tree (CART) analyses. In addition to aggregate VIS, CART analyses also included other predictors such as patient demographics, and highest lactate. Individuals who died were censored from the primary outcome analyses of length of stay and length of invasive mechanical ventilation. Pearson correlation coefficients were calculated for hospital length of stay and the aggregate VIS at multiple time points to analyze the correlation of these two variables over time. Receiver operator curves (ROC) were created to determine the optimal threshold for using average VIS AUC to predict mortality. Statistical significance was defined as a two-sided *p*-value <0.05. Commercially-available statistical software used included SAS v9.4 (SAS Institute, Inc., Cary, NC), IBM SPSS Statistics v26.0 (Armonk, NY: IBM Corp), and CART for the decision trees (Predictive Modeler) v8.3.2 (Salford Systems, San Diego, CA).

## Results

A total of 371 subjects were identified with a diagnosis of sepsis and a vasoactive medication requirement during the study period. All inclusion criteria were met by 199 eligible subjects with a median age of 7.2 years. Fifty-six percent of subjects were admitted from the CW Emergency Department and 19% from the acute care floors. Additional descriptive statistics summarizing the subjects are found in Table 1.

**Table 1:** Demographic and clinical characteristics of patients with severe sepsis included in the study.

**Demographics (*****n*** **=** **199)**	
**Age in years, median (IQR)**	7.2 (1.4, 13.4)
**Female**, ***n*** **(%)**	105 (53)
**Race**, ***n*** **(%)** White Black/African American (AA) Asian Other or Multi-race (Asian and AA) Not reported	– 130 (65.4) 43 (21.6) 10 (5.0) 5 (2.5) 11 (5.5)
**Ethnicity**, ***n*** **(%)** Not Hispanic/Latino Hispanic/Latino Not reported	– 158 (79.4) 34 (17.1) 7 (3.5)
**Patients source**, ***n*** **(%)** Emergency department (ED) Pediatric floor OSH transfer NICU Clinic Direct admission	– 113 (56.8) 37 (18.6) 37 (18.6) 8 (4.0) 2 (1.0) 2 (1.0)
**Clinical variables**	
**PRISM – III score, median (IQR)**	8 (3, 13)
**Lactate (mg/dL), median (IQR)**	27 (15, 49)
**Mortality**, ***n*** **(%)**	24 (12.1)
**Cardiac arrest**, ***n*** **(%)**	21 (10.6)
**ECMO**, ***n*** **(%)** Duration (days), median (IQR)	19 (9.5) 6.1 (2.7, 12.6)
**CRRT**, ***n*** **(%)** Duration (days), median (IQR)	18 (9.1) 9.4 (4.9, 14.5)

*IQR, interquartile range; ECMO, extracorporeal membrane oxygenation; CRRT, continuous renal replacement therapy*.

Primary outcome analyses were performed on all subjects discharged alive. For hospital LOS and PICU LOS, this included 175 (87.9%) of the 199 eligible subjects. Of the 124 subjects that were mechanically ventilated, 101 (81.4%) subjects were discharged alive and included in the analysis of mechanical ventilation days. The median hospital LOS was 12.3 days and median PICU LOS was 6.1 days (Table 2). One eligible patient was excluded from the mechanical ventilation cohort as an outlier as he/she was only mechanically ventilated for 0.1 days. The median mechanical ventilation days for the remaining cohort was 6.7 days (Table 2). A higher aggregate VIS was found to be associated with longer hospital LOS (*r* = 0.34, *p* < 0.0001), PICU LOS (*r* = 0.36, *p* < 0.0001), and increased mechanical ventilation days (*r* = 0.23, *p* = 0.018) (Table 2).

**Table 2:** Aggregate vasoactive-inotropic score (VIS) at 96h of hemodynamic drug support and its correlation with primary and secondary outcomes.

**Primary outcomes at 96 h following time 0**			
**Discharged alive (*****n*** **=** **176)**	**Median (IQR)**	**Pearson's correlation with aggregate VIS (r)**	***p*** **-value** ^**∧**^
Hospital LOS	12.3 (6.0, 30.5)	0.34	<0.0001^*^
PICU LOS	6.1 (2.2, 14.7)	0.36	<0.0001^*^
MV Days^a^	6.7 (3.1, 12.8)	0.23	0.018^*^
**Primary outcomes at 48 h**			
**VIS at 48 h and discharged alive (*****n*** **=** **65)**	**Median (IQR)**	**Pearson's correlation with VIS at 48 h (r)**	***p*** **-value** ^**∧**^
Hospital LOS	25.8 (12.1, 60.6)	0.36	0.003^*^
PICU LOS	14.4 (7.9, 39.2)	0.34	0.005^*^
MV Days^b^	7.9, (4.9, 28.8)	0.32	0.02^*^
**Secondary outcomes at 96 h following Time 0**			
**Full cohort (*****n*** **=** **199)**	**Aggregate VIS at 96 h, Median (IQR)**		
	**Yes**	**No**	***p*** **-value** ^**#**^
In-hospital mortality	16.9 (7.0, 31.4)	5.0 (2.8, 8.3)	<0.0001^*^
In-hospital cardiac arrest	7.4 (5.8, 14.4)	5.0 (2.8, 8.9)	0.006^*^
ECMO	20.7 (8.0, 31.1)	4.9 (2.8, 8.3)	<0.0001^*^
CRRT	21.0 (8.4, 29.7)	5.0 (2.9, 8.3)	<0.0001^*^
**Secondary outcomes at 48 h**			
**VIS at 48h (*****n*** **=** **82)**	**Aggregate VIS at 48 h, Median (IQR)**		
	**Yes**	**No**	***p*** **-value** ^**#**^
In-hospital mortality	17.0 (3.0, 21.5)	5.0 (2.0, 8.0)	0.058
In-hospital cardiac arrest	7.8 (5.0, 16.8)	5.0 (2.0, 8.5)	0.077
ECMO	17.0 (7.5, 24.0)	4.0 (2.0, 8.0)	<0.0001^*^
CRRT	18.0 (8.0, 23.0)	4.0 (2.0, 7.5)	0.0002^*^

*VIS, vasoactive inotrope score; IQR, interquartile range; LOS, Length of stay; MV, mechanical ventilation; ECMO, extracorporeal membrane oxygenation; CRRT, continuous renal replacement therapy*.

a *Analysis of mechanical ventilation days completed on the 102 subjects who were mechanically ventilated and discharged alive*.

b *Analysis of mechanical ventilation days completed on the 53 subjects who were mechanically ventilated, had VIS at 48h, and discharged alive*.

∧ *Pearson's correlation test performed to examine the relationship between the log transformed aggregate VIS and the related outcome variable*.

# *Mann-Whitney statistical test performed comparing the aggregate VIS between the Yes and No categories*.

* *Statistically significant p-values were < 0.05*.

Secondary outcome analyses of in-hospital mortality, in-hospital cardiac arrest requiring chest compressions, need for ECMO, and need for CRRT were performed on the full cohort of 199 subjects. Twenty-four (12.1%) subjects suffered in-hospital mortality, 21 (10.6%) had in-hospital cardiac arrest, 19 (9.5%) underwent ECMO cannulation, and 18 (9%) were on CRRT (Table 1). Of the 19 subjects cannulated onto ECMO, 18 (94.7%) were placed on veno-arterial (VA) ECMO and 1 (5.3%) placed on veno-venous (VV) ECMO. A total of 4 (2%) subjects suffered in-hospital cardiac arrest, underwent ECMO cannulation, and ultimately in-hospital mortality. A total of 3 (1.5%) subjects had in-hospital cardiac arrest and underwent ECMO cannulation but were discharged alive. The aggregate VIS was significantly higher in non-survivors (*p* < 0.0001), in the group that had in-hospital cardiac arrest (*p* = 0.006), required ECMO (*p* < 0.0001), or CRRT (*p* < 0.0001) (Table 2).

**Figure 1:**
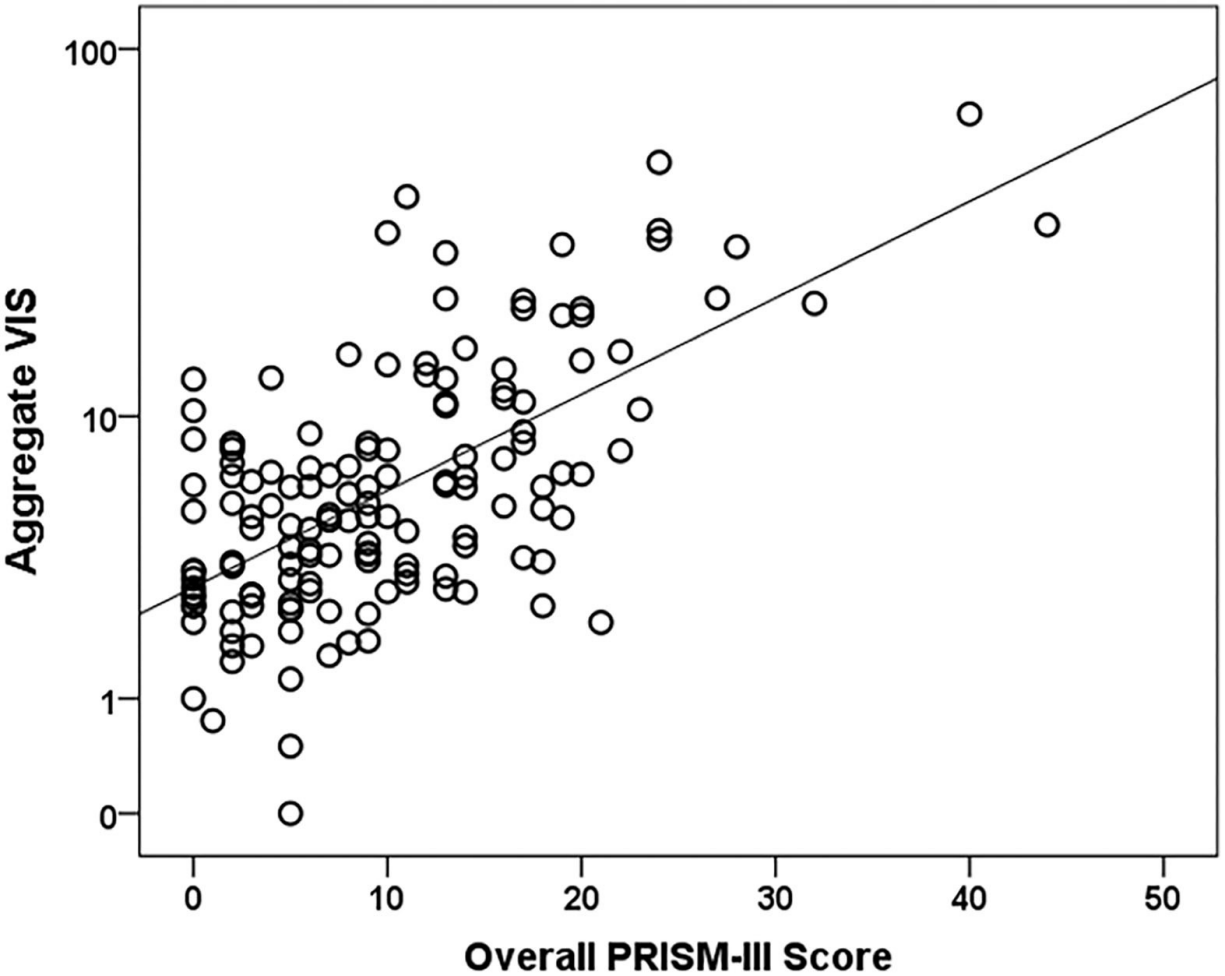
Aggregate VIS vs. PRISM-III severity of illness score scatter plot. *N* = 156. A Pearson's correlation test was used to compare the log of Aggregate VIS to PRISM-III scores (*r* = 0.6, *p*-value < 0.0001).

PRISM-III severity of illness scores incorporate physiologic data over the first 24 h of the pediatric ICU stay (14). Of the 199 eligible subjects, 156 (78.4%) subjects had a Time 0 with the initiation of vasoactive medications before or within 24 h of their PICU admission. These 156 subjects had VIS data to compare to PRISM-III scores, while the remaining 43 (21.6%) subjects were excluded as they did not have VIS data within the first 24 h of their PICU admission. A higher aggregate VIS was also associated with higher PRISM-III scores (*r* = 0.61, *p* < 0.0001) and increased risk of mortality percentages (*r* = 0.52, *p* < 0.0001). The scatter plot demonstrates positive correlation between aggregate VIS and PRISM-III scores, which is significantly different from zero (Figure 1).

Time point thresholds were calculated for the outcomes hospital LOS and mortality. The VIS score was most closely correlated to hospital LOS at hour 24 (Pearson's correlation coefficient = 0.38), but the correlation was statistically significant at every time point measured from hour 6 to 96 (Supplementary Table 3). ROC analysis demonstrated that average VIS AUC was most predictive of mortality at h 96 (Supplementary Figure 2). Individual hourly VIS was predictive of mortality as early as hour 2.

Data were also analyzed for VIS at 48 h to compare our study to previously published literature (11). Of our 199 subjects, 82 (41.2%) subjects had a VIS at 48 h, 65 (79.3%) of the 82 subjects were discharged alive, and 53 (81.5%) of the 65 subjects were mechanically ventilated. Subjects with a higher VIS at 48 h had longer hospital LOS (*r* = 0.36, *p* = 0.003), PICU LOS (*r* = 0.34, *p* = 0.005), and increased mechanical ventilation days (*r* = 0.32, *p* = 0.02) (Table 2). When analyzing secondary outcomes on the cohort of 82 subjects, 17 (20.7%) suffered in-hospital mortality, 12 (14.6%) had in-hospital cardiac arrest, 19 (23.2%) received ECMO, and 17 (20.7%) received CRRT. Higher VIS at 48 h was associated with increased need for ECMO (*p* < 0.0001) and need for CRRT (*p* = 0.0002) (Table 2).

**Figure 2:**
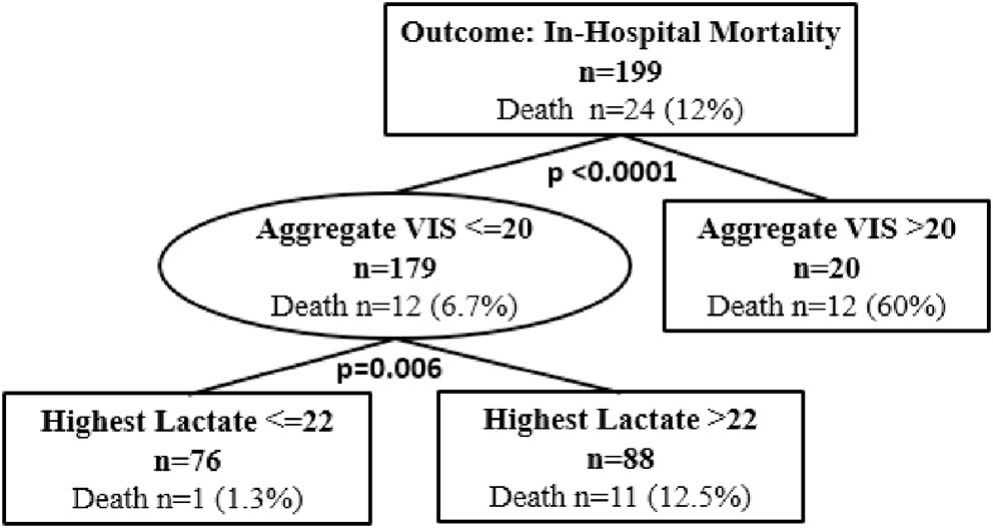
Classification and regression tree (CART) analysis for in-hospital mortality. *N* = 199. 15 subjects did not have a lactate drawn during the hospitalization. In CART analysis missing values are handled by the software substituting surrogate splitters.

**Figure 3:**
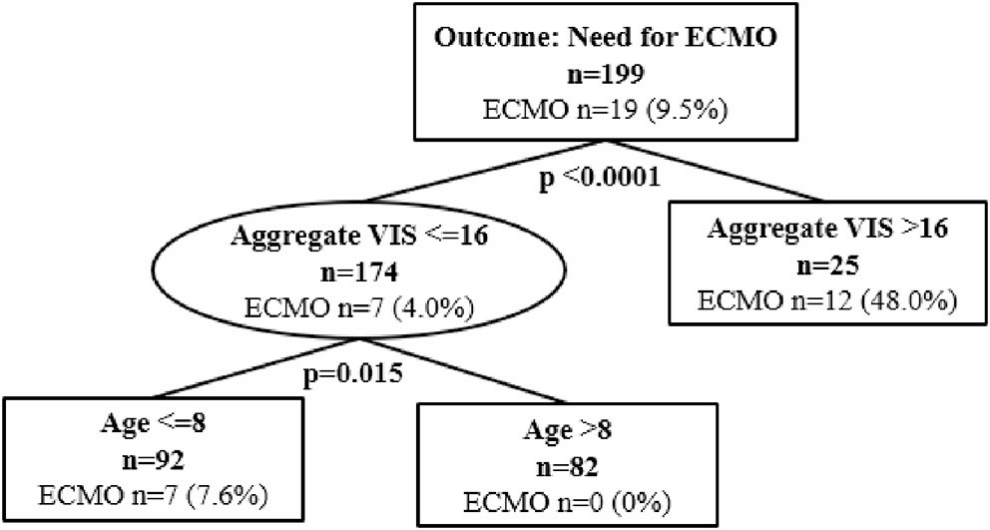
Classification and regression tree (CART) analysis for need for ECMO cannulation. *N* = 199. ECMO, Extracorporeal Membrane Oxygenation.

Classification and regression tree analyses were performed to identify risk factors associated with in-hospital mortality and ECMO cannulation. Predictors included in the CART analyses were aggregate VIS, patient demographics, and highest lactate level. At Children's Wisconsin, lactate is measured in mg/dL and the normal lactate level range is 4–15 mg/dL. Of the total subjects, 16 (8%) did not have a lactate value during their hospitalization. Twenty-four subjects (12.1%) died during their hospitalization and aggregate VIS was found to be an independent predictor for in-hospital mortality with a threshold of 20 (*p* < 0.0001) (Figure 2). For subjects with an aggregate VIS of ≤ 20, those with a highest lactate level of >22mg/dL were more likely to suffer in-hospital mortality (Figure 2). Nineteen subjects were managed with ECMO, 18 received VA-ECMO and 1 received VV-ECMO, and aggregate VIS was found to be an independent predictor for need for ECMO with a threshold of 16 (*p* < 0.0001) (Figure 3). For subjects with an aggregate VIS ≤ 16, those ≤ 8 years of age were more likely to receive ECMO (Figure 3).

CART analyses were also utilized to determine risk factors associated with mechanical ventilation days and included the same predictors as the other CART analyses. Of the 199 subjects, 102 (51.3%) subjects required mechanical ventilation and subjects age = 0 on admission were more likely to have increased mechanical ventilation days (*p* = 0.002) (Supplementary Figure 3). These subjects included 4 (3.9%) admitted to the NICU immediately after birth and subsequently transferred to the PICU during their hospitalization and 1 subject (1%) transferred from an outside facility immediately after birth with congenital heart disease. For subjects with age >0, those with highest lactate > 90 mg/dL had increased mechanical ventilation days (*p* = 0.008). For subjects with age >0 and highest lactate <= 90 mg/dL, aggregate VIS was an independent predictor of mechanical ventilation days with 16 as a possible threshold (*p* = 0.034) (Supplemental Figure 3).

## Discussion

This study attempts to demonstrate the possible benefits of aggregate VIS in predicting clinical outcomes in pediatric sepsis. Our study found that higher aggregate VIS was associated with longer LOS in both the PICU and hospital (*p* < 0.0001). Aggregate VIS was also significantly higher in non-survivors, subjects suffering an in-hospital cardiac arrest, and those requiring both ECMO and CRRT. Although we evaluated aggregate VIS retrospectively, there is potentially a benefit to utilizing aggregate VIS in real-time. The aggregate VIS thresholds established *via* CART analyses for in-hospital mortality (>20) and need for ECMO (>16) could be utilized to identify high-risk patients. Haque et al. demonstrated that patients with a high VIS (>20) had increased mortality rates (10). Our data analyses also identified an aggregate VIS >20 as an independent predictor for in-hospital mortality, complementing their study. This identification may aid clinicians in risk stratifying patients' disease states and affect ongoing management plans that directly impact morbidity. Clinicians may potentially be able to intensify or de-escalate certain aspects of patient care based on the risk stratification, such as central access, ventilatory support, enteral nutrition, sedative utilization, and/or rehabilitation.

Our study complements the single-center study from Colorado that examined VIS at a 48 h time point for all primary outcomes (11). McIntosh et al. did not find a statistically significant association between VIS at 48 h and death, cardiac arrest, or ECMO, which was attributed to a low frequency of outcomes and underpowered analysis (11). The study from Colorado did find a strong, independent association between VIS at 12 h and death, cardiac arrest, or ECMO (11). Our study also evaluated VIS at 48 h and found a significant association with need for ECMO, but not with in-hospital mortality or in-hospital cardiac arrest. When comparing aggregate VIS to VIS at 48 h in our study, aggregate VIS was found to be associated with all clinical outcomes and may be a better predictive tool for pediatric sepsis.

Although found to be statistically significant, the relationship between aggregate VIS and all primary outcomes, hospital LOS, PICU LOS, and mechanical ventilation days, had a low correlation coefficient with r ranging from 0.23–0.36. Additional multi-center studies need to be performed to better understand these associations. Of the 199 eligible patients, only 19 patients required ECMO with 18 using a VA configuration and 1 using VV configuration. Patients receiving VA ECMO may have a significant decrease in the VIS after cannulation, but this may not indicate that the patient's clinical condition was less severe. We analyzed aggregate VIS before and after ECMO cannulation, but these data were not statistically significant given the small sample size. Also, the lack of a statistically significant change in aggregate VIS before and after ECMO cannulation may indicate that ECMO is not frequently a curative treatment for sepsis. The analysis of involving ECMO and CRRT was further confounded by the fact that most individuals were not started on these therapies after the 96-h mark. Only one patient that required ECMO and nine patients that required CRRT had therapy initiated after 96 h. Most individuals began these therapies 24–48 h after time 0. The CART analysis partially accounts for this issue by using the aggregate VIS at the time of the event or at 96 h if the event was after that point in time compared to time 0.

There are several limitations in this study. The study was completed as a single-center retrospective study, focusing on a single institution's practice regarding sepsis management. VIS may be affected by institutional practices and policies regarding use of vasoactive medications and dosing. Additionally, Time 0 was established at the initiation of vasoactive medications instead of the onset of severe sepsis identification as this is difficult to identify retrospectively, which may have affected a patient's 96-h aggregate VIS, specifically when vasoactive medications were not initiated immediately. Laboratory data were not differentiated for origin (arterial, venous, capillary), anatomic location, or method of collection (tourniquet utilized or not), which may slightly skew values such as highest lactate levels. Furthermore, there are many clinical factors that can impact a patient's VIS. Not all patients were in an isolated septic shock state, which could affect their vasoactive medication needs and duration of support. In addition, management of other ICU interventions such as mechanical ventilation and sedation can affect a patient's cardiovascular dysfunction and thus have the potential to impact vasoactive medication titration/use.

## Conclusions

The data demonstrates a statistically significant association between aggregate VIS and many important clinical outcomes. The VIS score significantly correlated with hospital LOS at every time point between 6 and 96 h and the aggregate VIS was most predictive of mortality at 96 h. The VIS is an easy to calculate score that can be calculated at the bedside in any care setting or incorporated into the electronic medical record. This knowledge may allow clinicians to utilize aggregate VIS as a physiologic indicator to more accurately predict disease severity/trajectory in pediatric severe sepsis.

## Data Availability Statement

The raw data supporting the conclusions of this article will be made available by the authors, without undue reservation.

## Ethics Statement

The studies involving human participants were reviewed and approved by Children's Wisconsin Institutional Review Board. Written informed consent from the participants' legal guardian/next of kin was not required to participate in this study in accordance with the national legislation and the institutional requirements.

## Author Contributions

NT conceptualized and designed the study, obtained local IRB approval and revised/reviewed all drafts of the manuscript. PS conceptualized and designed the study, collected study data, and drafted the initial version of the manuscript. TP conceptualized and designed the study and revised the manuscript. LZ and KY performed statistical analysis and revised the manuscript. All authors approved the final version of the manuscript.

## Conflict of Interest

The authors declare that the research was conducted in the absence of any commercial or financial relationships that could be construed as a potential conflict of interest.

## Publisher's Note

All claims expressed in this article are solely those of the authors and do not necessarily represent those of their affiliated organizations, or those of the publisher, the editors and the reviewers. Any product that may be evaluated in this article, or claim that may be made by its manufacturer, is not guaranteed or endorsed by the publisher.

## Supplementary Material

Supplementary Figure 1 Vasoactive-inotrope score (VIS) calculation (9).

Supplementary Figure 2 Receiver Operator Curve (ROC) of VIS at hour 96 to predict Mortality. Area under the curve = 0.803.

Supplementary Figure 3 Classification and regression tree analysis for mechanical ventilation days.

Supplementary Table 1 Severe sepsis criteria from the International Consensus Conference on Pediatric Sepsis (4).

Supplementary Table 2 Inclusion and exclusion criteria. ^*^Time 0 is the onset of vasoactive medications.

Supplementary Table 3 Relationship of Aggregate VIS to Hospital Length of Stay at Various Time Points.
