# Supplementary figures and images for: Using Aggregate Vasoactive-Inotrope Scores to Predict Clinical Outcomes in Pediatric Sepsis
Source: Front Pediatr. 2022 Mar 4;10:778378. doi: 10.3389/fped.2022.778378 (PMC8931266; doi:10.3389/fped.2022.778378)

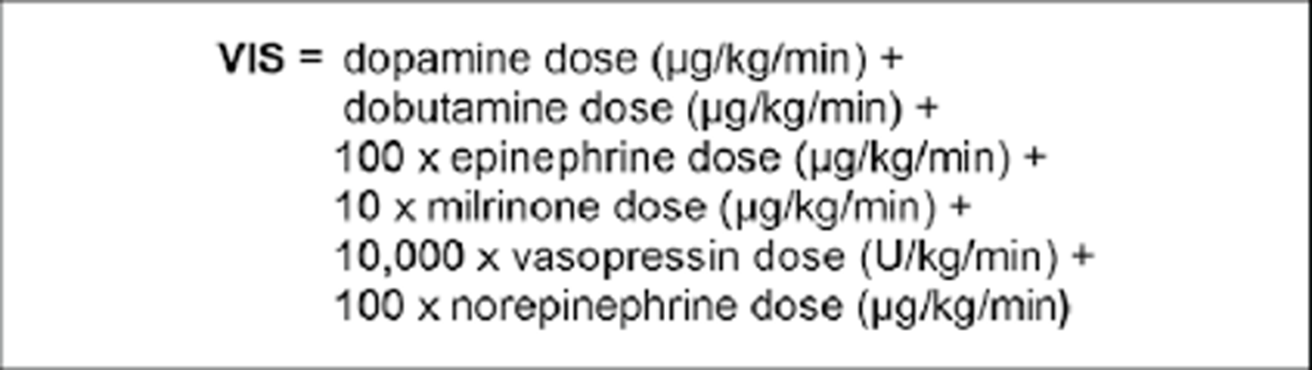

Supplement: Supplementary Figure 1 — Vasoactive-inotrope score (VIS) calculation (9). [file Image_1.TIFF]

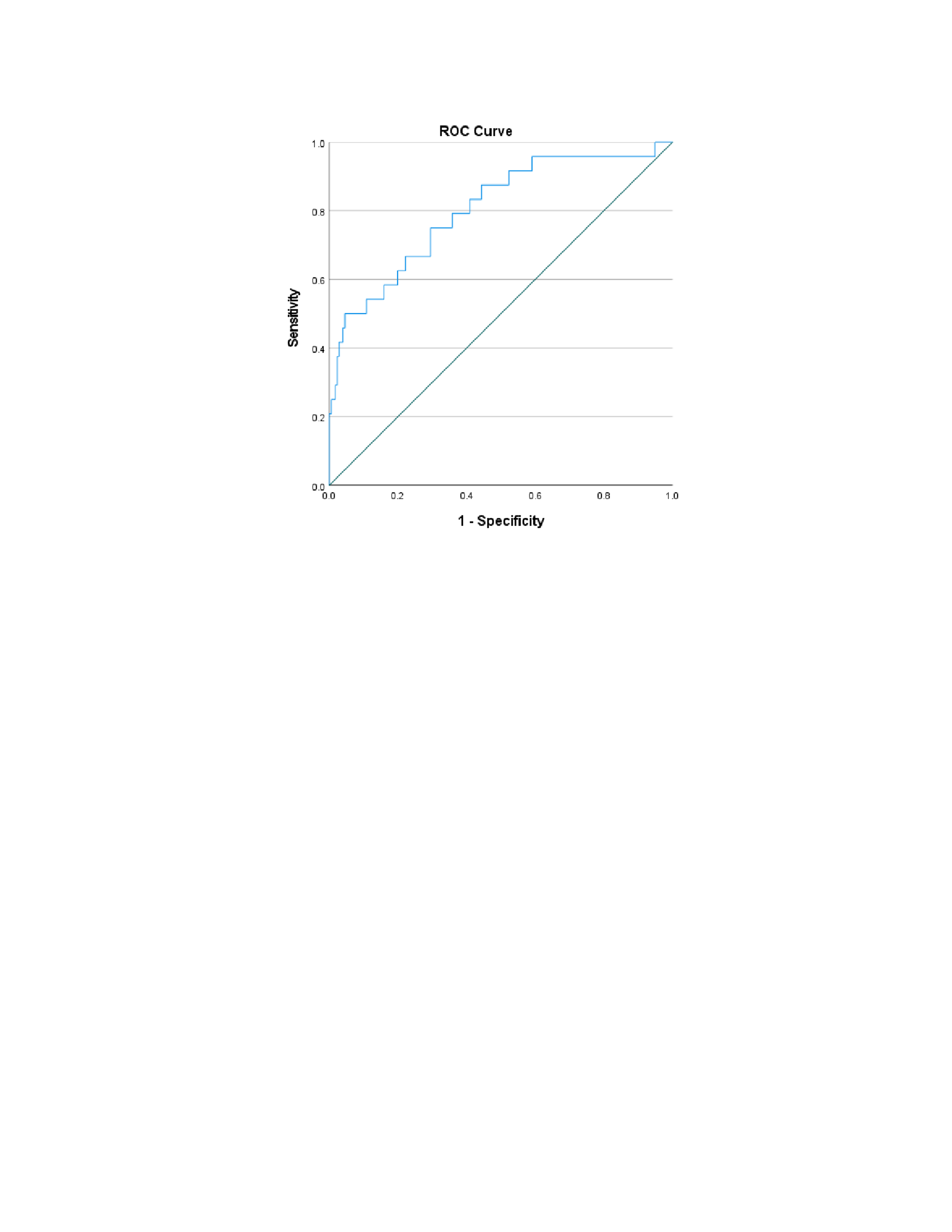

Supplement: Supplementary Figure 2 — Receiver Operator Curve (ROC) of VIS at hour 96 to predict Mortality. Area under the curve = 0.803. [file Image_2.TIFF]

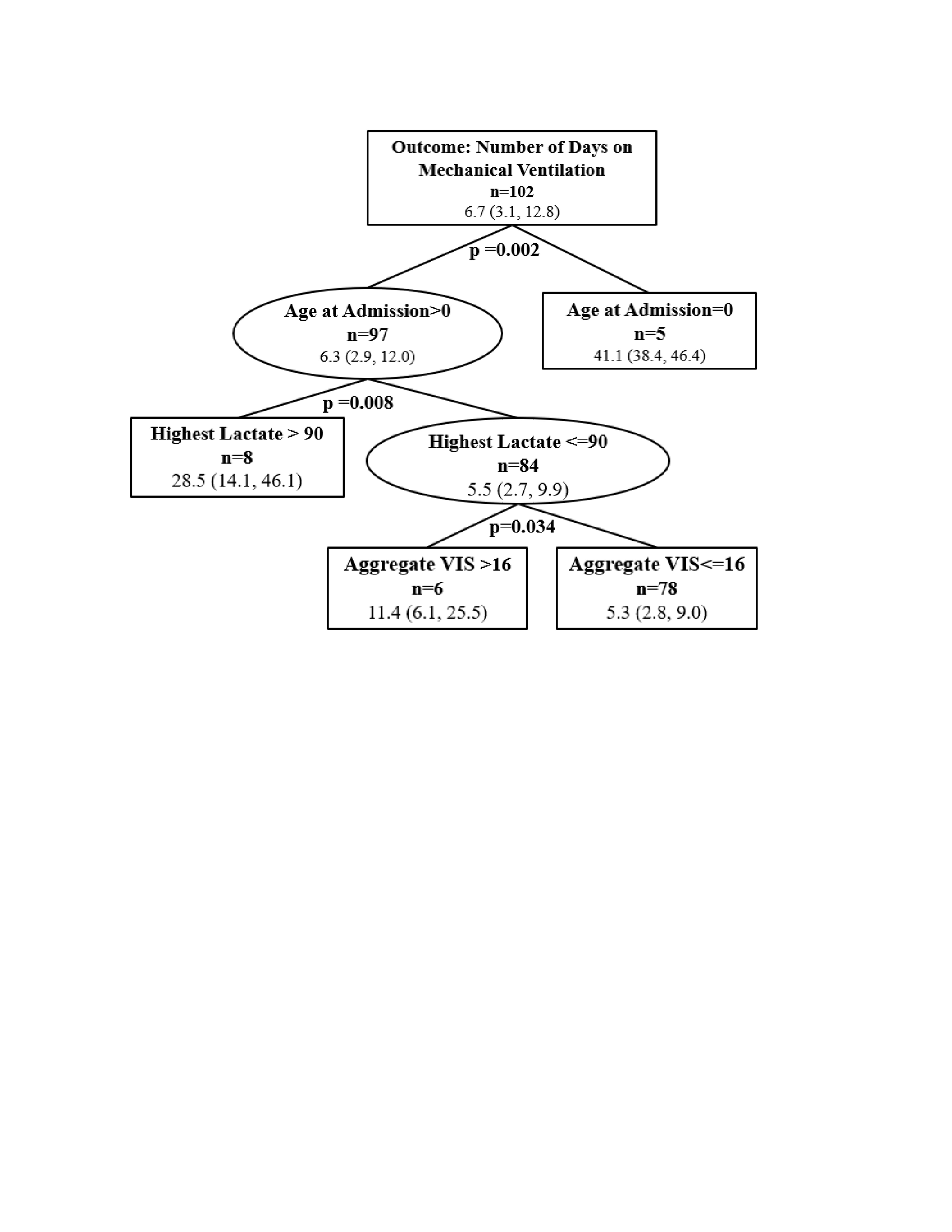

Supplement: Supplementary Figure 3 — Classification and regression tree analysis for mechanical ventilation days. [file Image_3.tiff]
