# Supplementary material for: Using Aggregate Vasoactive-Inotrope Scores to Predict Clinical Outcomes in Pediatric Sepsis
Source: Front Pediatr. 2022 Mar 4;10:778378. doi: 10.3389/fped.2022.778378 (PMC8931266; doi:10.3389/fped.2022.778378)
Supplement: Supplementary Table 1 — Severe sepsis criteria from the International Consensus Conference on Pediatric Sepsis (4). [file Table_1.docx]

Supplemental Table 1: Severe sepsis criteria from the International Consensus Conference on Pediatric Sepsis [4].

| **Organ System** | **Criteria** |
| --- | --- |
| **Cardiovascular** | Hypotension  *-OR-*  Reliance on a vasoactive drug to maintain blood pressure  *-OR-*  (2) or more of the following:   - Metabolic acidosis that cannot be explained by another cause such as ketoacidosis, intoxication, etc. - Elevated arterial lactate - Oliguria - Prolonged capillary refill |
| **Respiratory** | Acute respiratory distress syndrome (ARDS)  *-OR-*  Arterial CO_2_ > 65 or 20mm Hg over baseline  Proven need for > 50% FiO_2_ to maintain SpO_2_ ≥ 92%  *-OR-*  Need for non-elective invasive or non-invasive mechanical ventilation |
| **Neurologic** | Glascow coma score ≤ 11  *-OR-*  Acute change in mental status |
| **Hematologic** | Platelet count < 80,000/microL (or a decline of 50% from highest value recorded over the last 3 days for chronic hematology/oncology patients)  *-OR-*  Disseminated intravascular coagulation (DIC) |
| **Renal** | Serum creatinine ≥ 2 times upper limit of normal for age or two-fold increase in baseline creatinine |
| **Hepatic** | Total bilirubin ≥ 4mg/dL (not applicable to newborn)  *-OR-*  Alanine aminotransferase (ALT) > 2 times upper limit of normal for age |
