# Supplementary material for: Using Aggregate Vasoactive-Inotrope Scores to Predict Clinical Outcomes in Pediatric Sepsis
Source: Front Pediatr. 2022 Mar 4;10:778378. doi: 10.3389/fped.2022.778378 (PMC8931266; doi:10.3389/fped.2022.778378)
Supplement: Supplementary Table 2 — Inclusion and exclusion criteria. *Time 0 is the onset of vasoactive medications. [file Table_2.DOCX]

| **Inclusion Criteria** | **Exclusion Criteria** |
| --- | --- |
| Age 0 – 17 years at the time of diagnosis | Age ≥ 18 years at the time of diagnosis |
| Pediatric ICU admission | Exclusive neonatal ICU admission |
| Time 0* on/after January 1, 2017 and sepsis treatment completed before/on July 31, 2019 | Time 0* before January 1, 2017 and/or sepsis treatment completed after July 31, 2019 |
| Initial encounter for critical sepsis, severe sepsis, or septic shock during a single hospitalization | Subsequent diagnoses of critical sepsis, severe sepsis, or septic shock episodes during the same hospitalization or subsequent hospitalizations after the initial encounter |
| Received vasoactive medications for a sepsis diagnosis | No vasoactive medications or vasoactive medications for a non-sepsis diagnosis |

Supplemental Table 2: Inclusion and exclusion criteria. *Time 0 is the onset of vasoactive medications
