# Supplementary material for: Using Aggregate Vasoactive-Inotrope Scores to Predict Clinical Outcomes in Pediatric Sepsis
Source: Front Pediatr. 2022 Mar 4;10:778378. doi: 10.3389/fped.2022.778378 (PMC8931266; doi:10.3389/fped.2022.778378)
Supplement: Supplementary Table 3 — Relationship of Aggregate VIS to Hospital Length of Stay at Various Time Points. [file Table_3.docx]

Relationship of Aggregate VIS to Hospital Length of Stay at Various Time Points

| **Time Point** | **Correlation Coefficient (r)** | **p-value** |
| --- | --- | --- |
| Hour 6 | 0.291 | < 0.0001 |
| Hour 12 | 0.330 | < 0.0001 |
| Hour 24 | 0.376 | < 0.0001 |
| Hour 36 | 0.369 | < 0.0001 |
| Hour 48 | 0.361 | < 0.0001 |
| Hour 60 | 0.350 | < 0.0001 |
| Hour 72 | 0.341 | < 0.0001 |
| Hour 84 | 0.342 | < 0.0001 |
| Hour 96 | 0.343 | < 0.0001 |

Comparison of Aggregate VIS at each time point to hospital length of stay made using Pearson correlation test. N = 176. P-value of <0.05 considered statistically significant
